# Boosting Visible‐Light Photocatalytic Redox Reaction by Charge Separation in SnO_2_/ZnSe(N_2_H_4_)_0.5_ Heterojunction Nanocatalysts

**DOI:** 10.1002/chem.202000468

**Published:** 2020-07-20

**Authors:** Yeonho Kim, Dong‐Won Jeong, Jaewon Lee, Min Young Song, Sang Moon Lee, Jihoon Choi, Du‐Jeon Jang, Hae Jin Kim

**Affiliations:** ^1^ Division of Analytical Science Korea Basic Science Institute Daejeon 34133 Republic of Korea; ^2^ Department of Chemistry Seoul National University Seoul 08826 Republic of Korea; ^3^ Department of Material Science and Engineering Chungnam National University Daejeon 34134 Republic of Korea; ^4^ Research Institute of Basic Sciences Incheon National University Incheon 22012 Republic of Korea

**Keywords:** charge separation, environmental pollutants, heterojunctions, reactive oxygen species, visible-light photocatalysis

## Abstract

In this work, environmentally friendly photocatalysts with attractive catalytic properties are reported that have been prepared by introducing SnO_2_ quantum dots (QDs) directly onto ZnSe(N_2_H_4_)_0.5_ substrates to induce advantageous charge separation. The SnO_2_/ZnSe(N_2_H_4_)_0.5_ nanocomposites could be easily synthesized through a one‐pot hydrothermal process. Owing to the absence of capping ligands, the attached SnO_2_ QDs displayed superior photocatalytic properties, generating many exposed reactive surfaces. Moreover, the addition of a specified amount of SnO_2_ boosted the visible‐light photocatalytic activity; however, the presence of excess SnO_2_ QDs in the substrate resulted in aggregation and deteriorated the performance. The spectroscopic data revealed that the SnO_2_ QDs act as a photocatalytic mediator and enhance the charge separation within the type II band alignment system of the SnO_2_/ZnSe(N_2_H_4_)_0.5_ heterojunction photocatalysts. The separated charges in the heterojunction nanocomposites promote radical generation and react with pollutants, resulting in enhanced photocatalytic performance.

## Graphical abstract

**Effective nanocatalysts**: Nontoxic SnO_2_/ZnSe(N_2_H_4_)_0.5_ nanocatalysts were easily synthesized through a one‐pot hydrothermal process and showed excellent photocatalytic performance under visible light. The enhancement in the photocatalytic activity originates from efficient charge separation within a distinct type II band structure.

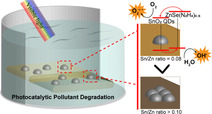

## Introduction

Owing to the continuous release of vast amounts of industrial pollutants globally, wastewater treatment processes are of significant importance to mankind. Among the advanced oxidation processes (AOPs) reported to date, the photocatalytic technique based on a polymer, metal, and semiconductor has shown great potential as an efficient and green process to help mitigate these environmental problems.^1^, ^2^, ^3^, ^4^ Semiconductor photocatalysts have been studied for several decades, owing to their superior material properties, including the higher absorption cross‐section and structural stabilities, compared to those of organic materials. These materials offer efficient ways of capturing light for energy harvesting, such as photovoltaic and photocatalytic hydrogen production, using unlimited solar power.^2^, ^5^, ^6^, ^7^, ^8^, ^9^, ^10^, ^11^ Additionally, diverse methods have been developed to modify the band gaps of these semiconductors.^3^, ^12^ These reasons make semiconductors great candidates as future photocatalysts that can decompose hazardous organic compounds.^4^, ^13^

Unfortunately, the large bandgaps of these materials and relatively short exciton lifetime make the use of excited excitons in semiconductor applications very challenging.^14^, ^15^, ^16^ For example, TiO_2_ is a well‐known photocatalyst but only responds in the ultraviolet region. This limits their availability to undergo redox reactions with pollutants, significantly decreasing their efficiency.^16^, ^17^, ^18^ Compared to the single‐component semiconductor TiO_2_, heterojunction semiconductors with a type II band structure prolong the lifetime of excitons.^15^, ^19^, ^20^, ^21^ The combination of semiconductors with different compositions and core/shell structures results in different reduction/oxidation potentials, thereby allowing band gap engineering, electron‐hole pair separation, and surface activities. Thus, researchers have studied various heterojunction systems such as direct Z‐Scheme, p–n junction, type II system, and surface heterojunctions. These heterojunction systems have also been used in various applications fields including photocatalysts as well as electrocatalysts, supercapacitors, and lithium batteries.^22^, ^23^, ^24^, ^25^, ^26^, ^27^, ^28^, ^29^, ^30^ Well‐defined and staggered band structures can facilitate the spatial separation of excitons as well as redox reaction on their surfaces, thereby boosting the generation of radical species in the reaction solution.^3^

The production of efficient photocatalysts for industrial application depends on a facile and energy‐efficient synthetic method as well as the catalytic performance of the catalyst.

Inorganic–organic hybrid nanocomposites comprising a semiconductor material and layer‐by‐layer‐assembled organic linker molecules have the advantage of being able to be handled like a polymer, even though they display semiconductor properties.^31^, ^32^ Various types of ligands having at least one functional group on each side can be used as linkers and thus, diamine species have been studied thoroughly.^33^ Hybrid nanocomposites can be easily synthesized from these ligands and semiconductor precursors. Moreover, the use of inorganic–organic hybrid nanocomposites as photocatalysts has been studied because of their facile synthesis as well as their superior performance compared to that of semiconductor materials.^31^, ^33^, ^34^ The process mechanism has not been entirely unraveled; however, it has been suggested that nitrogen‐containing amine groups modify the semiconductor charge distribution, so that organic dye pollutants can easily approach the nanocomposite by partially charged electrostatic interactions.^35^, ^36^ However, much research on the catalytic mechanism and catalyst performance of inorganic–organic hybrid nanocomposites is still required.

In this study, we focused on the facile synthesis of a heterojunction material with efficient charge separation ability. Here, we present the synthesis of SnO_2_/ZnSe(N_2_H_4_)_0.5_ heterojunction nanocatalysts. Using the distinct quasi‐one‐dimensional substrate of the inorganic–organic hybrid nanocomposite ZnSe(N_2_H_4_)_0.5_, we have applied SnO_2_ quantum dots (QDs) on the nanocomposite as a photocatalytic mediator through a facile hydrothermal method within a one‐pot process. With this advantageous type II band alignment, the heterojunction nanocatalysts achieve enhanced charge separation, which was confirmed by photoluminescence (PL) spectra and decay kinetic profiles. The increased exciton lifetime increases the probability of reaction with pollutants and thus, enhances the photocatalytic performance of the heterojunction nanocatalysts. Notably, this system can only be applied to chemicals that respond to visible light. Both ZnSe(N_2_H_4_)_0.5_ and SnO_2_ exhibit a slight response in blue light; however, in this system, the electrons are mainly transferred from rhodamine B (RhB) to the heterojunction nanocomposite. Excited RhB undergoes N‐deethylation, and the complete mineralization of the rhodamine species occurs through radicals generated from the redox reaction between the nanocomposite and oxygen or water molecules. The redox reaction of the organic dye and spectroscopic data provide direct evidence that SnO_2_ QDs modify the nanocomposite band gap to type II band alignment, thereby enhancing the photocatalytic performance of the SnO_2_/ZnSe(N_2_H_4_)_0.5_ nanocatalysts.

## Results and Discussion

**Figure 1:**
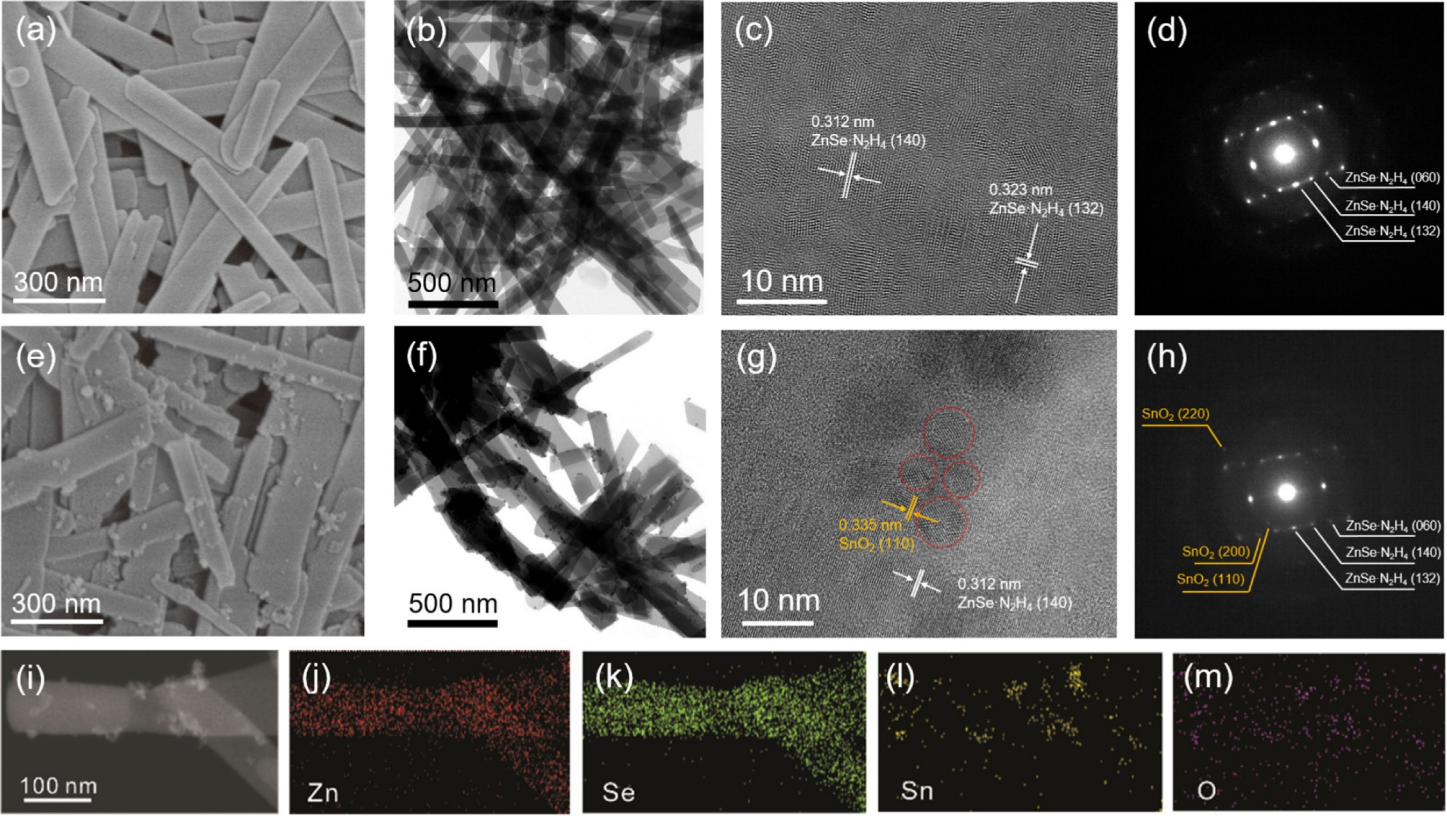
(a) SEM image, (b, c) TEM images, and (d) corresponding SAED pattern of the pristine ZnSe(N_2_H_4_)_0.5_. (e) SEM image, (f, g) TEM images, (h) corresponding SAED pattern, (i) STEM image, and (j–m) EDX elemental maps of SnO_2_/ZnSe(N_2_H_4_)_0.5_ heterojunction nanocomposites with a R_Sn/Zn_ value of 0.08.

Figures 1 a,b presents the respective typical scanning (SEM) and transmission (TEM) electron microscopy images of the hydrazine‐intercalated hybrid nanostructure of ZnSe(N_2_H_4_)_0.5_ nanobelts. The as‐obtained hybrid nanobelts had an average diameter of 200 nm and a typical length of a few micrometers. The high‐resolution TEM (HRTEM) image in Figure 1 c reveals lattice spacings of 0.312 and 0.323 nm, corresponding to the (140) and (132) plane spacings of the ZnSe(N_2_H_4_)_0.5_ structure, respectively. The corresponding selected area electron diffraction (SAED) patterns in Figure 1 d indicate the preferred growth orientation of the ZnSe(N_2_H_4_)_0.5_ nanostructure to the *b*‐axis. Moreover, the (132) and (140) facets corresponding to ZnSe(N_2_H_4_)_0.5_ support the quasi‐one‐dimensional structure of the ZnSe(N_2_H_4_)_0.5_ nanobelts.^34^ The SnO_2_ quantum dots (QDs) were directly dressed onto the surface of ZnSe(N_2_H_4_)_0.5_ to fabricate the nanocomposite photocatalysts, SnO_2_/ZnSe(N_2_H_4_)_0.5_, via a second hydrazine‐assisted hydrothermal process using a one‐pot synthetic method (Figure 1 e,f). The quasi‐one‐dimensional morphology of ZnSe(N_2_H_4_)_0.5_ was retained even after the second hydrothermal process. The weight percentage of the SnO_2_ QDs on the ZnSe(N_2_H_4_)_0.5_ nanobelts was controlled by varying the concentration of the tin precursor. Thus, the density of the SnO_2_ QDs increased with increasing Sn/Zn ratio (R_Sn/Zn_), while the size of the SnO_2_ QDs (≈4.1 nm) remained almost constant (Figure S1). The marked red circles in the HRTEM image (Figure 1 g) indicate that the well‐crystallized SnO_2_ QDs were successfully fabricated onto the ZnSe(N_2_H_4_)_0.5_ nanobelts. The measured lattice spacing, calculated as 0.335 nm, corresponded to the (110) plane spacing of the bulk rutile SnO_2_ structure.^19^ The SAED pattern (Figure 1 h) revealed the coexistence of diffractions from both SnO_2_ and ZnSe(N_2_H_4_)_0.5_, indicating the formation of the heterojunction nanocomposite rather than the alloy composite.^3^ The scanning TEM (STEM) image and energy dispersive X‐ray (EDX) elemental analysis (Figure 1 i–m) revealed the homogenous distribution of the SnO_2_ QDs onto the whole ZnSe(N_2_H_4_)_0.5_ surface. However, with increasing R_Sn/Zn_ value, the dressed SnO_2_ QDs tended to aggregate due to their bare surface (i.e., without ligand capping; Figure S2). The HRTEM image in Figure S3 also illustrates the aggregating nature of the SnO_2_ QDs with increasing R_Sn/Zn_. However, in this case, the sizes of the individual SnO_2_ QDs were near‐identical (3.9 nm). The EDX elemental data (Figure S4) revealed calculated atomic Sn/Zn ratios of 0.03, 0.05, 0.07, and 0.11 for the samples with R_Sn/Zn_=0.03, 0.05, 0.08, and 0.10, respectively. These values implied that in the high‐yield oxidation process, nearly all the added tin precursor was converted to SnO_2_ on ZnSe(N_2_H_4_)_0.5_ via a second hydrothermal process in a one‐pot method.

**Figure 2:**
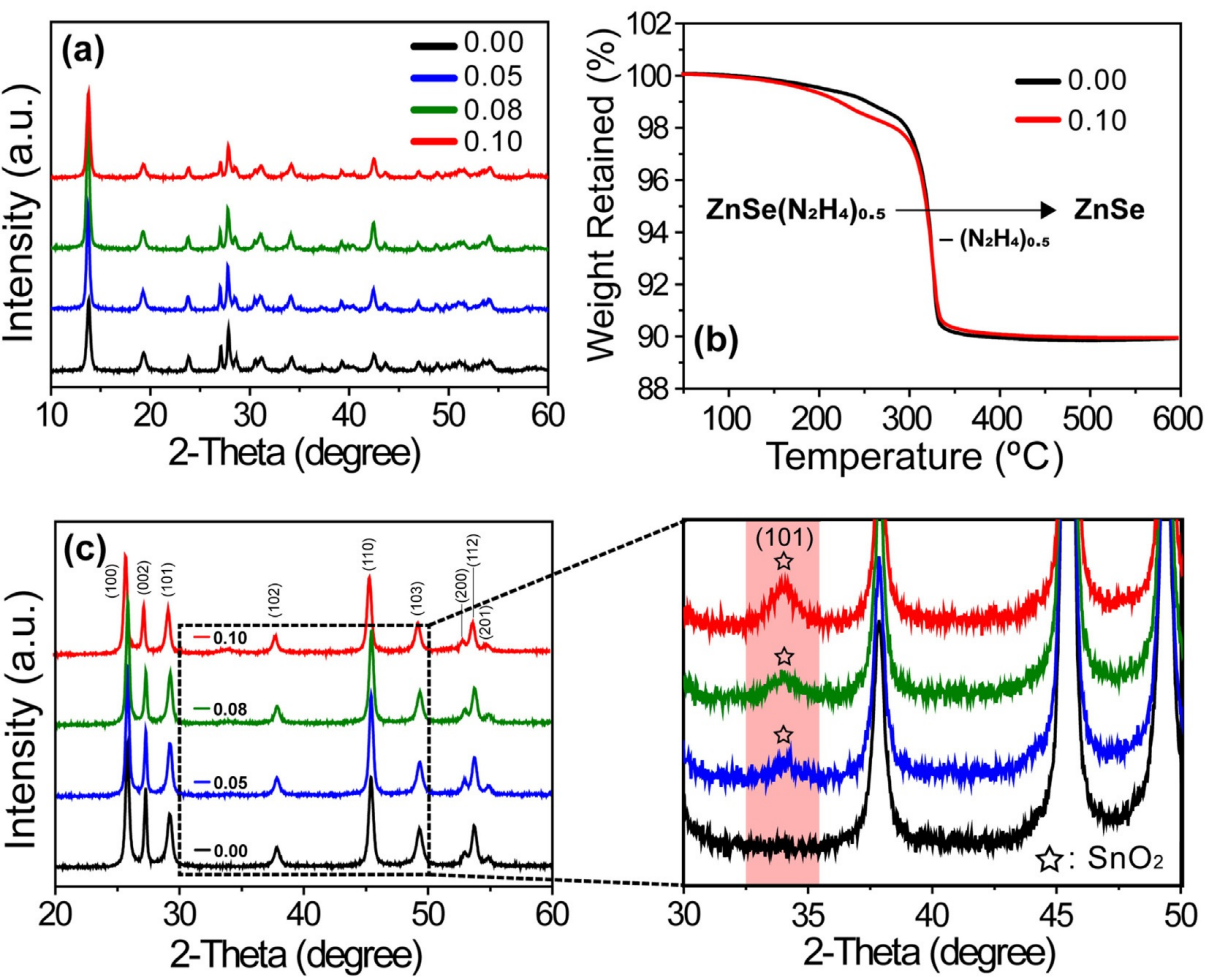
(a) XRD and (b) TGA of SnO_2_/ZnSe(N_2_H_4_)_0.5_ heterojunction nanocomposites with indicated R_Sn/Zn_ values. (c) XRD analysis and their enlarged views of SnO_2_/ZnSe fabricated via thermal treatment, showing the existence of SnO_2_ quantum dots on ZnSe surfaces.

The as‐synthesized heterojunction nanocatalysts were characterized by X‐ray diffraction (XRD) and thermogravimetric (TG) analysis. All the XRD patterns (Figure 2 a) revealed the presence of a prominent organic‐amine related diffraction peak at 13.8°, corresponding to a *d*‐spacing of 6.41 Å.^32^, ^34^ Considering that the monolayer thickness of ZnSe is 2.12 Å (from 2*θ*=42.5°), the interlayer distance was estimated as 4.29 Å. The organic spacer in the hybrid nanostructure was analyzed by TG experiments. The curves in Figure 2 b reveal an 11.3 % weight loss, from 200 to 400 °C, with decomposition mainly occurring at approximately 280 °C. These results are consistent with the theoretical content of hydrazine in ZnSe(N_2_H_4_)_0.5_.^3^, ^34^ Notably, even when dressing SnO_2_ onto the ZnSe(N_2_H_4_)_0.5_ nanobelts for the SnO_2_/ZnSe(N_2_H_4_)_0.5_ (R_Sn/Zn_=0.10) nanocomposites, the weight loss and curve shape remained almost identical to those observed for ZnSe(N_2_H_4_)_0.5_ (R_Sn/Zn_=0.00). TG analysis also implied that all the hydrazine molecules only bonded to Zn metal and that the added Sn^4+^ precursor was completely converted to SnO_2_, without binding organic spacer of hydrazine, to form Sn(N_2_H_4_) and SnO_2_(N_2_H_4_) complexes. After thermal treatment, the ZnSe(N_2_H_4_)_0.5_ nanobelts were converted to pure wurtzite

ZnSe nanostructures (Figure 2 c).^37^, ^38^ To clarify the existence of SnO_2_, we conducted thermal annealing of ZnSe(N_2_H_4_)_0.5_ with various R_Sn/Zn_ values at 600 °C under N_2_ atmosphere for 4 h. The SEM images (Figure S5) indicated that the samples maintained their one‐dimensional morphologies even after thermal treatment, and the obtained ZnSe product matched the reference wurtzite ZnSe (JCPDS card no. 80‐008).^39^ The enlarged image in the right panel of Figure 2 c describes the diffraction peak at about 34°, confirming the growth of rutile SnO_2_ nanocrystals.^3^ The broad and weak peak revealed a small grain size (≈6.5 nm), which was calculated by Scherrer's equation.^19^, ^40^ Possibly, the overgrowth of SnO_2_ grains was still retained during the thermal annealing process. Moreover, the change in R_Sn/Zn_ did not significantly affect the ZnSe crystal structure, while the peak intensity at about 34°, originating from rutile SnO_2_, increased with increasing R_Sn/Zn_.

We conducted RhB redox experiments to evaluate the photocatalytic activities of the as‐synthesized heterojunction nanocomposites. Although electrochemical analysis is a powerful analytical tool, organic dye degradation experiments can still provide intuitive evaluation results of the photocatalytic activity.^41^, ^42^, ^43^, ^44^, ^45^, ^46^ In these experiments, we employed visible light (*λ*≥400 nm) from an incident Xe lamp with cut‐off filters to eliminate the direct decomposition effect of RhB by UV light.

**Figure 3:**
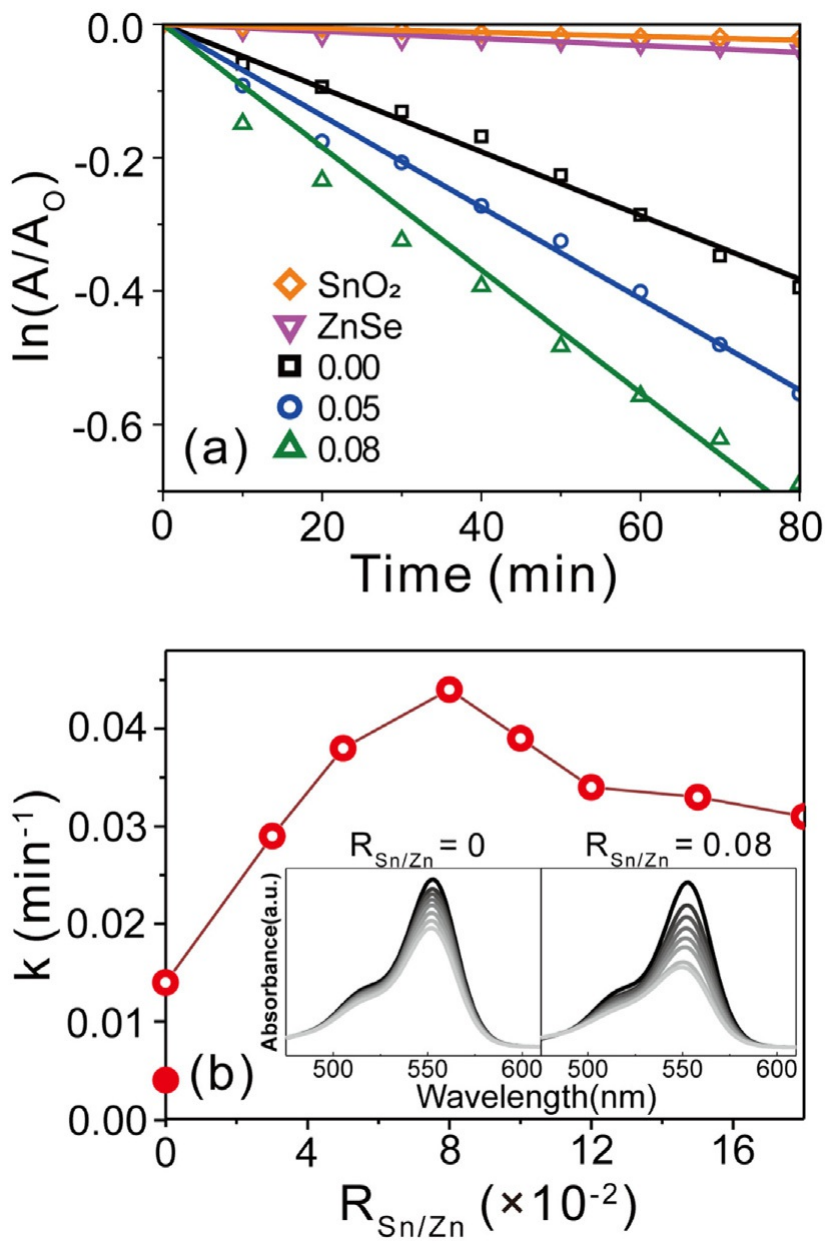
(a) First‐order decay profiles and (b) degradation rate constants of RhB in terms of R_Sn/Zn_ values (hollow red dots) with pristine ZnSe (solid red dots). The inset shows that the absorption of RhB decreases with time.

The catalytic performance of the materials was evaluated by pseudo‐first‐order kinetics (Figure 3 a), which revealed that the RhB concentrations decreased with increasing R_Sn/Zn_. At the microscopic level, when the RhB species are excited by visible light, they pass electrons to ZnSe(N_2_H_4_)_0.5_. Because of the redox potential difference between ZnSe(N_2_H_4_)_0.5_ and SnO_2_, the electrons then move to SnO_2_ to generate radicals that decompose and mineralize the RhB species. We observed that ZnSe(N_2_H_4_)_0.5_ exhibited a superior performance to the intrinsic performances of the ZnSe nanoparticles and SnO_2_ (Table S1). However, we concluded that further research was needed to understand why this behavior occurs, especially in ZnSe(N_2_H_4_)_0.5_. Figure 3 b compares the rate constants of the nanocomposites with various R_Sn/Zn_ values. The as‐synthesized ZnSe(N_2_H_4_)_0.5_ nanocomposite that did not comprise SnO_2_ displayed a superior catalytic performance than pristine ZnSe and outperformed the performance of the commercial catalyst P25 (Figure 3 a and Figure S6).^34^ Notably, the catalytic performance reached a maximum at the R_Sn/Zn_ value 0.08. The insets in Figure 3 b also indicate that the absorbance of the nanocomposite with R_Sn/Zn_=0.08 decreased at a faster rate than that of the R_Sn/Zn_=0.00 nanocomposite. This is because the SnO_2_ QDs attached to ZnSe(N_2_H_4_)_0.5_ induce charge separation. Thus, the excited RhB becomes oxidized by transferring electrons to ZnSe(N_2_H_4_)_0.5_ and subsequently, the electrons flow from ZnSe(N_2_H_4_)_0.5_ into the SnO_2_ QDs. This charge separation therefore increases the possibility of photocatalytic reaction with the adsorbed oxygen and water molecules.

With the further increase in R_Sn/Zn_ (>0.08) however, a decline in the photocatalytic performance was observed. We noticed that porosimetry analysis could not explain this phenomenon because there was no significant difference in the total pore volume. This is illustrated in Figure S7 and Table S1, where the total pore volumes did not change much more than the rate constants and the BET surface area and average pore size of the samples remained constant. Thus, we concluded that the surface pore distribution was not the key to explain how the photocatalytic performance changes occurred, and further analysis was necessary to understand the peak point of the photocatalytic performance. We therefore proceeded to elucidate the optimized conditions of our catalytic system and perform spectroscopic analyses to propose the working mechanism.

**Figure 4:**
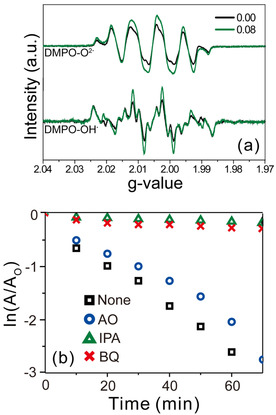
(a) EPR spectra of the radicals generated from SnO_2_/ZnSe(N_2_H_4_)_0.5_ heterojunction nanocomposite with indicated RSn/Zn values, and (b) first‐order decay profiles of RhB by SnO_2_/ZnSe(N_2_H_4_)_0.5_ (R_Sn/Zn_=0.08) heterojunction nanocomposites in the presence of various radical scavengers.

To justify whether the photocatalytic activity mechanism of the as‐synthesized heterojunction nanocomposite is driven by radicals, electron paramagnetic resonance (EPR) spectroscopy was used to directly detect the radicals. Both the superoxide (O_2_^.−^) and hydroxyl (^.^OH) radicals were presented in the EPR data (Figure 4 a) in the form of 5,5‐dimethyl‐1 pyrroline *N*‐oxide (DMPO) adducts, indicating that the heterojunction nanocomposite generated radicals. In addition, attachment of the SnO_2_ QDs increased radical generation. This was consistent with the results obtained from the photocatalytic experiment, which revealed that more radicals were generated from the heterojunction nanocomposite, and supported the efficient photocatalytic activity of SnO_2_/ZnSe(N_2_H_4_)_0.5_.

Radical scavengers are a useful tool to analyze whether radical species drive the reaction, and we used *p*‐benzoquinone (BQ), isopropanol (IPA), and ammonium oxalate (AO) for the investigation (Figure 4 b). In general, AO is considered to act different from BQ and IPA; AO, which is an oxalate, is advantageous for inner‐sphere electron transfer, whereas BQ and IPA follow an outer‐sphere electron transfer regime.^47^ Excitons are generated when the incident light is irradiated onto the sample and scavengers proceed through the reaction. At this step, BQ and IPA barely approach the surface and thus, they do not accept electrons directly from the nanocomposite. As time goes by, the separated excitons create radicals that attack BQ and IPA to react.^48^, ^49^ In the case of AO, the carboxylate groups assist the scavenger to approach the surface of the nanocomposite so that it can receive holes directly from the semiconductor instead of reacting with radicals. Notably scavengers cannot distinguish individual radical species because the superoxide generated from the electrons can subsequently be converted into hydrogen peroxide and the hydroxyl radical. The RhB absorbance changes confirm that BQ (8.0 %) and IPA (5.4 %) almost halt the reaction, while AO (83.3 %) only has a slight effect on the rate of the reaction compared to that of the R_Sn/Zn_=0.08 sample (100 %) without any scavenging molecules (Table S2). Since the BQ and IPA scavenging radicals influence the reaction as compared to AO, it was suggested that radicals are the main cause of the photocatalytic reaction of the as‐synthesized nanocatalysts. Additionally, EPR data supports that photocatalytic activity occurs in the heterojunction nanocomposite via radicals (Figure 4 a). Thus, using the acquired data, we suggested the following photocatalytic activity mechanism: an electron from RhB transfers after the molecule becomes excited, and the heterojunction nanostructure generates radicals^17^, ^50^, ^51^, ^52^, ^53^ (Figure S8). In the case of RhB*^+^, *N*‐deethylation occurs on the surface (Figure S8 c) of the nanostructures, illustrated by the blueshifted absorption peaks in Figure S9. The deethylated species (*N*,*N*‐diethyl‐N’‐ethylrhodamine, *N*‐ethyl‐N’‐ethylrhodamine, *N*‐ethylrhodamine, and rhodamine, where some molecules have isomers) exhibit relatively weak absorption. However, rhodamine, which does not contain an ethyl group, has a molar extinction coefficient of 8.4×10^4^ m ^−1^ cm^−1^, as compared to the 11.5×10^4^ m ^−1^ cm^−1^ of RhB, which is significant.^54^ In Figure S9, the absorption spectra of RhB in the presence of light with the R_Sn/Zn_=0.08 sample displayed an almost quenched absorbance after 70 min, with the species having an absorption peak centered at ≈500 nm. Thus, our data revealed the occurrence of deethylation as well as mineralization. In addition, previous studies have revealed that deethylated species can boost mineralization. Therefore, the decrease in the visible absorption of RhB indicates that complete mineralization progressed with the as‐synthesized heterojunction nanostructure. We also conducted a reusability test to confirm the structural stability of our catalytic system (Figure S10). Thus, SnO_2_/ZnSe(N_2_H_4_)_0.5_ with R_Sn/Zn_=0.08 was used for RhB degradation and, after the fifth test, the rate constant was 16.8 % of that observed in the first test. Normally, heterojunction semiconductor materials have an advantage on charge separation because the two different semiconductors have different redox potentials, thus causing a spatial difference. Instead, charge imbalance is more likely to occur in our proposed system, and the structures become unstable after photocatalytic reaction. In fact, the results revealed that the nanocomposite efficiency plummets with reuse, as was previously reported.^3^, ^34^ These results indicate that further studies are needed to improve the structural stability of future photocatalysts.

**Figure 5:**
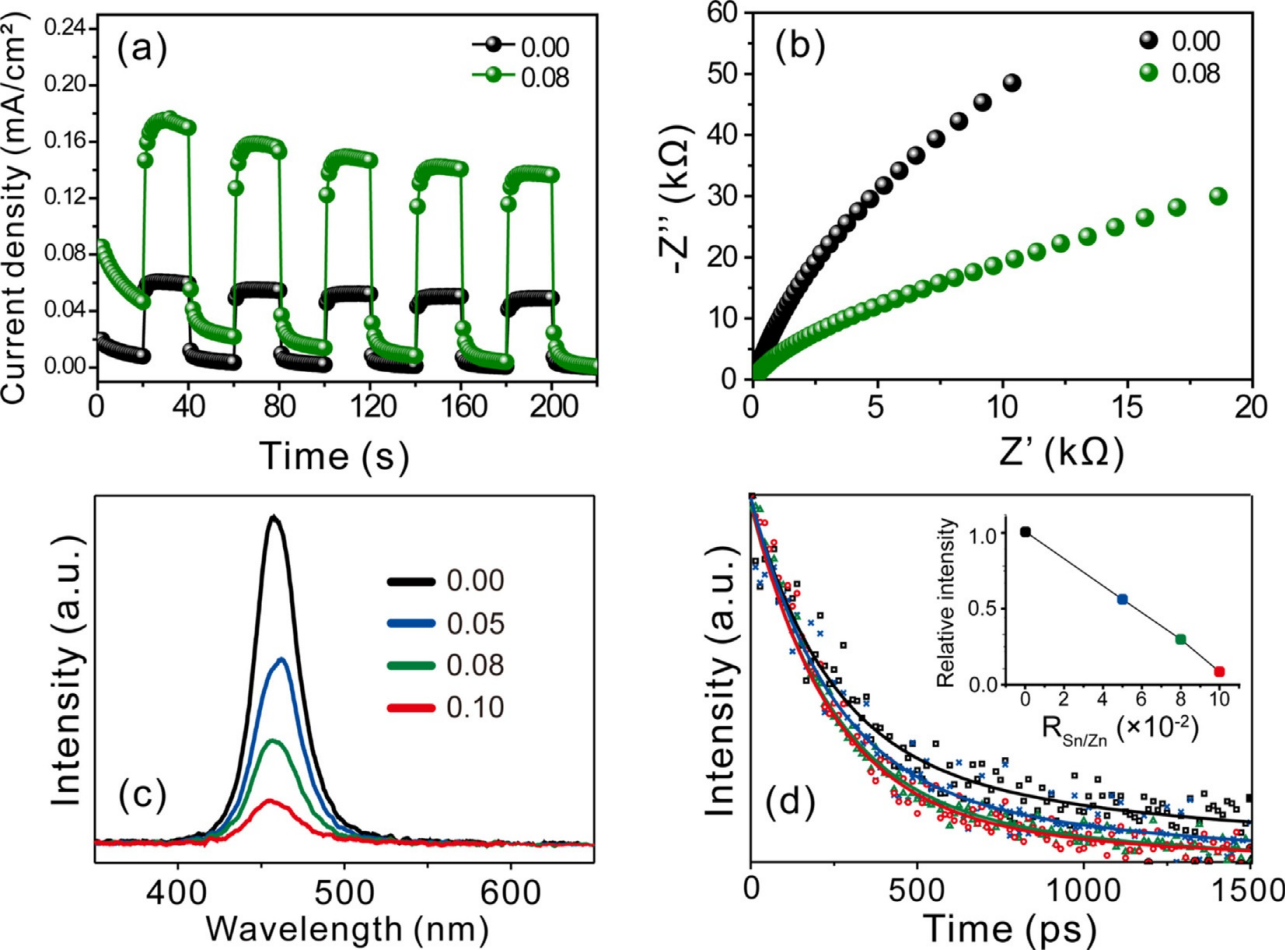
(a) Transient photocurrent response curves, (b) electrochemical impedance spectra, (c) PL spectra, and (d) PL kinetics of interfacial charge transfer of SnO_2_/ZnSe(N_2_H_4_)_0.5_ heterojunction nanocomposites with indicated R_Sn/Zn_ values.

Figure 5 a displays the photoelectrochemical measurements to further reveal the transfer efficiency of the excitons. The photocurrent density of SnO_2_/ZnSe(N_2_H_4_)_0.5_ (R_Sn/Zn_=0.08) was 2.42‐fold greater (0.143 mA cm^−2^) than that of ZnSe(N_2_H_4_) (0.059 mA cm^−2^), indicating the suppressed recombination of electron‐hole pairs in the nanocomposites. The generated transient photocurrent gradually decreased during the repeated on/off cycles (Figure S11). Photocorrosion still occurred in our heterojunction photocatalysis system, results that are consistent with those of the reusability tests (Figure S10). We therefore supposed that the decreasing photocurrent density originated from electrolyte depletion as well as the dissolution of selenide ions into the electrolyte due to photocorrosion.^55^, ^56^ Electrochemical impedance spectroscopy (EIS; Figure 5 b) was also conducted to study the interfacial characteristics of the photocatalysts. As displayed in Figure 5 b, the SnO_2_/ZnSe(N_2_H_4_)_0.5_ nanocomposites displayed a smaller arc diameter than that of ZnSe(N_2_H_4_)_0.5_, indicating its lower charge‐transfer resistance. Spectroscopic data not only indicate the existence of chemicals but can also be used to prove mechanisms. Thus, the absorption spectra (Figure S12) confirmed the presence of SnO_2_ QDs in the nanocomposites,^3^ while the photoluminescence (PL) spectra and kinetics profiles suggested that these SnO_2_ QDs can also act as a great catalytic mediator. To clearly investigate the charge separation mechanism of the heterojunction nanostructure, we needed to directly excite the excitons from the valence band and observe the optical properties of the nanostructure. Thus, the excitation light with a wavelength of 266 nm, which is shorter than the wavelength corresponding to the ZnSe (N_2_H_4_)_0.5_ bandgap, was irradiated to study the charge separation mechanism in the hybrid nanostructure. Figure 5 c illustrates that the nanocomposite emitted quenched photoluminescence at 460 nm with the increase in R_Sn/Zn_.^34^ The full width at the half maximum (FWHM) of the PL spectra was approximately 35 nm, and the PLs originated from the band‐edge emission of ZnSe(N_2_H_4_)_0.5_. Considering that only a single sharp peak was observed regardless of the R_Sn/Zn_ value, we suggested that only ZnSe(N_2_H_4_)_0.5_ contributed to the PL, while the SnO_2_ QDs did not display any PL emission. Thus, we inferred that another non‐radiative pathway was generated by the attachment of the SnO_2_ QDs. The PL kinetic profiles (Figure 5 d) indicated that the exciton lifetimes of the nanocomposites decreased with increasing R_Sn/Zn_ value. Thus, while the fast component (Table S3) arose from laser scattering, the second component originated from the decay of the band‐edge emission of the nanocomposite. Therefore, the lifetime decrease of the second PL decay component with increasing R_Sn/Zn_ value justified that the SnO_2_ quantity was linearly related to the charge separation efficiency of the nanocomposite. These phenomena reveal that excitons are separated so that the electrons move into SnO_2_ while the holes remain in the ZnSe(N_2_H_4_)_0.5_ (Figure S13).

We surprisingly noted that the charge transfer efficiency increased steadily with increasing R_Sn/Zn_ value. On the other hand, the catalytic performance did not exhibit the same tendency, and the performance deteriorated when the R_Sn/Zn_ value exceeded 0.08 (Figure 3 b). We therefore supposed that the SnO_2_ surface area present on the nanocomposite played a crucial role in the photocatalytic activity. As previously mentioned, the total pore size or interfacial charge transfer efficiency cannot explain the optimum photocatalytic performance of the SnO_2_/ZnSe(N_2_H_4_)_0.5_ nanocomposite at R_Sn/Zn_=0.08. Instead, a reasonable explanation of the photocatalytic performance would be to consider the total surface area of the SnO_2_ QDs, on which the separated electrons may reside to react and generate radicals. Charge transfer occurs near the surface through adsorption or tunneling, so it is important to obtain a large surface area to improve the catalytic reactions.^47^ The EM images (Figures 1 g, Figures S3 and S5) illustrate that the R_Sn/Zn_=0.10 sample displays more aggregation in SnO_2_ than the R_Sn/Zn_=0.08 sample. This may cause a decrease in the exposed reactive surface of SnO_2_, thereby reducing the photocatalytic performance of the nanocomposite. Thus, to reduce the high surface energy, the aggregation behavior of SnO_2_ QDs is more likely to occur. We assumed that the aggregated SnO_2_ QDs on the ZnSe(N_2_H_4_)_0.5_ would negatively affect the photocatalytic performance because there is a relatively smaller exposed reactive surface of the aggregated SnO_2_ QDs with which the molecules can react to be finally converted into radicals.

**Figure 6:**
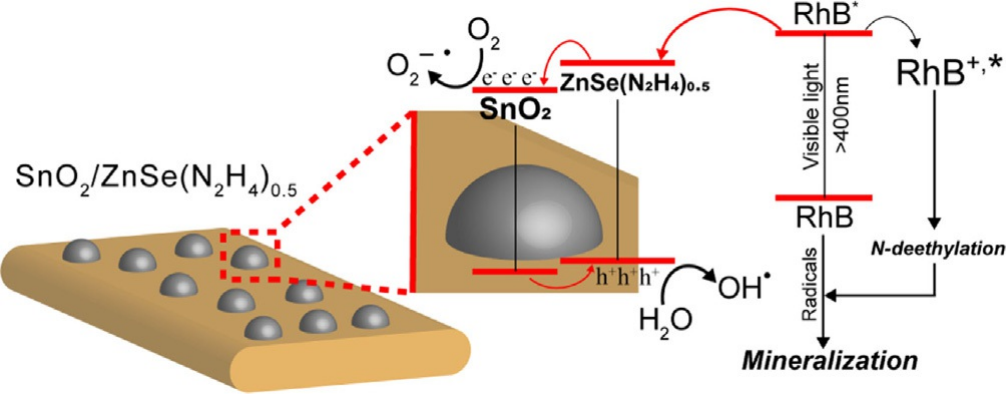
Schematic drawing of photocatalytic activities of a SnO_2_/ZnSe(N_2_H_4_)_0.5_ heterojunction nanocomposite. Reaction pathways in this drawing are exaggerated for simplicity.

From the results in this study we concluded that SnO_2_ QDs can effectively act as a catalytic mediator, which increases the charge separation efficiency of the ZnSe(N_2_H_4_)_0.5_ nanobelts (Figure 6). We also suggest that the decrease in the catalytic performance of the nanocomposites having R_Sn/Zn_>0.08 is caused by aggregation of the SnO_2_ QDs, which present a less exposed reactive surface.

## Conclusions

In summary, SnO_2_/ZnSe(N_2_H_4_)_0.5_ heterojunction nanocatalysts, prepared by controlling the dressing amounts of SnO_2_ quantum dots (QDs), were applied to photocatalytic pollutant degradation. To increase the catalytic properties, we fabricated well‐crystallized SnO_2_ QDs (≈4.2 nm) without capping ligands onto quasi‐one‐dimensional ZnSe(N_2_H_4_)_0.5_ nanobelts through a facile hydrazine‐assisted hydrothermal process. The optimized nanocatalyst (R_Sn/Zn_=0.08) presented 12.0‐ and 3.3‐fold enhanced performances compared to those of the commercial ZnSe and ZnSe(N_2_H_4_)_0.5_ nanobelts, respectively. The photocatalytic working mechanism was revealed by photoelectrochemical analytical data as well as photoluminescence spectra and decay kinetic profiles. The attached SnO_2_ QDs acted as a catalytic mediator and constructed the advantageous type II band alignment of SnO_2_/ZnSe(N_2_H_4_)_0.5_. Moreover, by controlling the attaching amounts of SnO_2_ QDs and suppressing the aggregating nature to boost the catalytic performance of the ZnSe(N_2_H_4_)_0.5_ nanobelts, the nanocatalysts were optimized by maximizing their exposed reactive surface to enhance their catalytic reactions. In consequence, the as‐synthesized nanocatalysts facilitated charge separation through a type II band structure, which increased the exciton lifetime and probability of the catalyzed reaction to occur. Thus, the proposed catalysts show great potential as heterojunction nanocatalysts that function in the visible‐light region.

## Experimental Section

**Materials**: All the chemicals were used as received without further purification: tin chloride trihydrate (98 %), zinc chloride (98 %), selenium (99.5 %), hydrazine monohydrate (98 %), ethylenediamine (99 %), *p*‐benzoquinone (BQ, 98 %), ammonium oxalate monohydrate (AO, 99 %), zinc selenide (99.99 %), 5,5‐dimethyl‐1pyrroline *N*‐oxide (DMPO, 97 %), titanium (IV) oxide (P25), and tin (IV) oxide; isopropyl alcohol (IPA, 99.5 %), methanol (99 %), and ethanol (99 %) from Daejung Chemicals; rhodamine B (RhB, s) from Wako Pure Chemical. Ultrapure deionized water (>18 MΩ cm) from a Millipore Milli‐Q system was used throughout the experiments.

**Preparation of ZnSe(N_2_H_4_)_0.5_ and SnO_2_/ZnSe(N_2_H_4_)_0.5_ hybrid nanocomposites**: For the preparation of the pristine ZnSe(N_2_H_4_)_0.5_ nanobelts, 2.0 mmol of Se into 20 mL of N_2_H_4_⋅H_2_O was added to 1.0 mmol of ZnCl_2_ into 20 mL of water with vigorous stirring for 30 min.^32^ The mixture was loaded into a Teflon‐lined stainless‐steel autoclave, placed in a preheated oven at 180 °C for 6 h, and then cooled to room temperature. A white precipitate produced in the reaction mixture was washed three times with water and ethanol, vacuum‐dried, and kept in a vial for further characterization. To attach SnO_2_ quantum dots onto ZnSe(N_2_H_4_)_0.5_ for the fabrication of SnO_2_/ZnSe(N_2_H_4_)_0.5_ hybrid nanocomposites, the proper amount of SnCl_4_⋅3 H_2_O was added into the above the ZnSe(N_2_H_4_)_0.5_ containing mixture and stirring for 20 min.^3^ Then, the mixture solution was poured into a Teflon‐lined stainless‐steel autoclave. Note that R_Sn/Zn_, the molar ratio of Sn to Zn in the final mixture, was varied from 0.00 to 0.12. The sealed autoclave was put again into a preheated oven at 180 °C for another 6 h. The resultant products were repeatedly centrifuged, washed with water and ethanol several times, and finally dried in a vacuum to obtain powdered SnO_2_/ZnSe(N_2_H_4_)_0.5_ hybrid nanocomposites.

**Characterization**: Transmission electron microscopy (TEM) and high‐angle annular dark‐field (HAADF) images with energy‐dispersive X‐ray (EDX) elemental mappings were recorded by a ZEISS Libra 200 HT Mc Cs (200 kV). Scanning electron microscopy (SEM) images were obtained with a Hitachi S4800 microscope while high‐resolution X‐ray diffraction (HRXRD) patterns were analyzed with a PANanalytical Empyrean multipurpose diffractometer (Cu‐Kα anode at 40 kV and 30 mA). Brunauer–Emmett–Teller (BET) specific surface area and pore size were determined by N_2_ adsorption–desorption with Micromeritics ASAP 2020. Thermogravimetric analysis (TGA) was conducted with a PerkinElmer Pyris 1 TGA at 50–600 °C and a heating rate of 10 °C min^−1^ in nitrogen flux. Electron paramagnetic resonance (EPR) spectra were measured by a Bruker EMX/Plus spectrometer equipped with a dual‐mode cavity (ER 4116DM) with microwave tuned at 9.13 GHz. DMPO was used for spin‐trapping agent. Detection of oxygen radicals and hydroxyl radicals was performed in methanol and water as solvents, respectively. UV/Vis absorption spectra were obtained with a Shimadzu UV‐2450 spectrophotometer and photoluminescence (PL) spectra were recorded using an Ocean Optics USB2000+ detector with excitation of 266 nm pulses from a Q‐switched Quantel Brilliant Nd:YAG laser. PL decay kinetic profiles were obtained with a Hamamatsu C2830 streak camera of 10 ps attached with a Princeton Instruments RTE128H CCD detector after exciting samples using 266 nm pulses from a mode‐locked Quantel Pizzicato ND:YAG laser of 25 ps. PL wavelengths were selected by combining cutoff and band‐pass filters.

**Photocatalytic performance**: The photocatalytic performances of composites nanocatalysts were measured by monitoring the photodegradation of rhodamine B (RhB). Detailed procedures of photocatalytic reaction are as follows: for each catalytic reaction, 30 mL of the mixed solution containing 5.0 mg nanocatalysts and 10 μm RhB was vigorously stirred in a dark room for 60 min to ensure the establishment of an adsorption–desorption equilibrium between the nanocatalysts and RhB molecules. The mixture solution was irradiated for different time intervals with the light beam of a 300 W Xenon lamp (Newport, model 66 902). The setup includes a water filter to cut the IR and cutoff filter (<400 nm) to cut ultraviolet light. Each aliquot was centrifuged to separate the supernatant, whose concentration of RhB solution was monitored by measuring UV/Vis absorption spectra with a spectrophotometer (Shimazu, UV‐2450). In order to evaluate the photostability of catalysts, all the experimental parameters were kept constant. After each reaction, the color of a mixture solution became colorless. Then, a fresh RhB solution was added into the resultant solution, where the initial concentration of RhB was always 10 μm. The experiments were repeated 5 times under the same conditions.

## Conflict of interest

The authors declare no conflict of interest.

## Supporting information

As a service to our authors and readers, this journal provides supporting information supplied by the authors. Such materials are peer reviewed and may be re‐organized for online delivery, but are not copy‐edited or typeset. Technical support issues arising from supporting information (other than missing files) should be addressed to the authors.

Supplementary
