# Supplementary material for: Boosting Visible‐Light Photocatalytic Redox Reaction by Charge Separation in SnO2/ZnSe(N2H4)0.5 Heterojunction Nanocatalysts
Source: Chemistry. 2020 Jul 20;26(46):10510–8. doi: 10.1002/chem.202000468 (PMC7496910; doi:10.1002/chem.202000468)
Supplement: Supplementary file 1 — Supplementary [file CHEM-26-10510-s001.pdf]

Chemistry–A European Journal

Supporting Information

Boosting Visible-Light Photocatalytic Redox Reaction by Charge Separation in $SnO_2/ZnSe(N_2H_4)_{0.5}$ Heterojunction Nanocatalysts

Yeonho Kim+,[a, d] Dong-Won Jeong+,[b] Jaewon Lee,[a, b] Min Young Song,[a] Sang Moon Lee,[a] Jihoon Choi,*[c] Du-Jeon Jang,*[b] and Hae Jin Kim*[a]

Structure characterization

Figure S1. TEM images.

Figure S2. STEM and EDX mapping images.

Figure S3. HRTEM image.

Figure S4. EDX spectra and their elemental analysis.

Figure S5. SEM images.

Catalytic performance

Table S1. Detailed information of BET analysis.

Figure S6. First-order decay profiles.

Figure S7. BET analysis.Photocatalytic performances.

Table S2. Catalytic degradation rate constants under various hole scavengers

Figure S8. Suggest mechanism of photocatalytic activity.

Figure S9. Absorption spectra of of RhB solutions during the photocatalytic reaction.

Figure S10. Reusability tests.

Optical characterization

Figure S11. Photocurrent stability test

Figure S12. Extinction spectra.

Table S3. Decay time constants.

Figure S13. Schematic drawing of charge transfer.

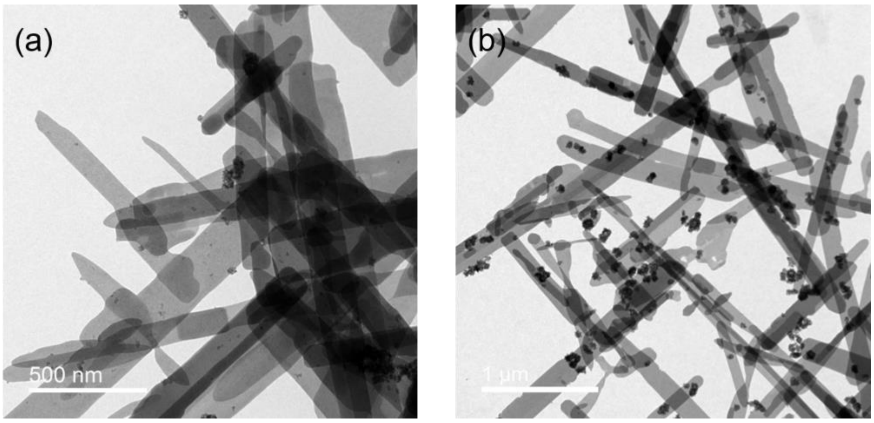

Figure S1. TEM images of $SnO_2/ZnSe(N_2H_4)_{0.5}$ heterojunction nanocomposites having $R_{Sn/Zn}$ = 0.03 (a) and 0.12 (b).

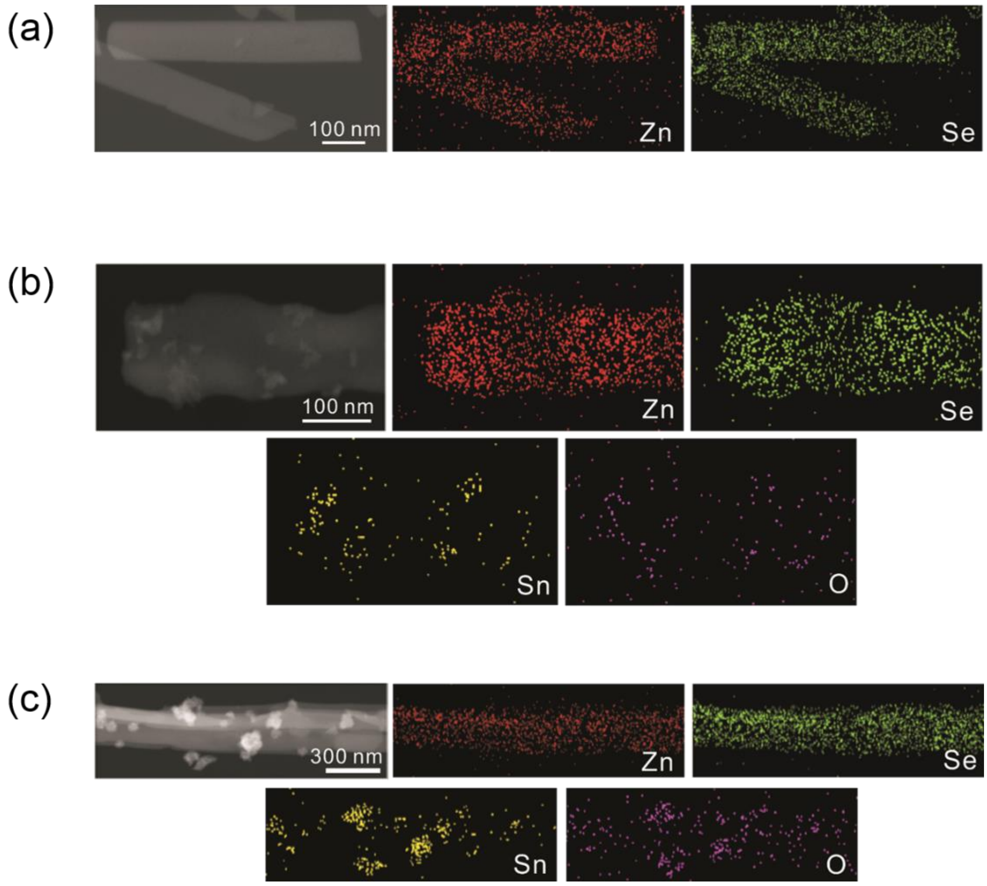

Figure S2. STEM images and EDX elemental mapping images of $SnO_2/ZnSe(N_2H_4)_{0.5}$ heterojunction nanocomposites having $R_{Sn/Zn}$ = 0.00 (a), 0.05 (b), and 0.10 (c).

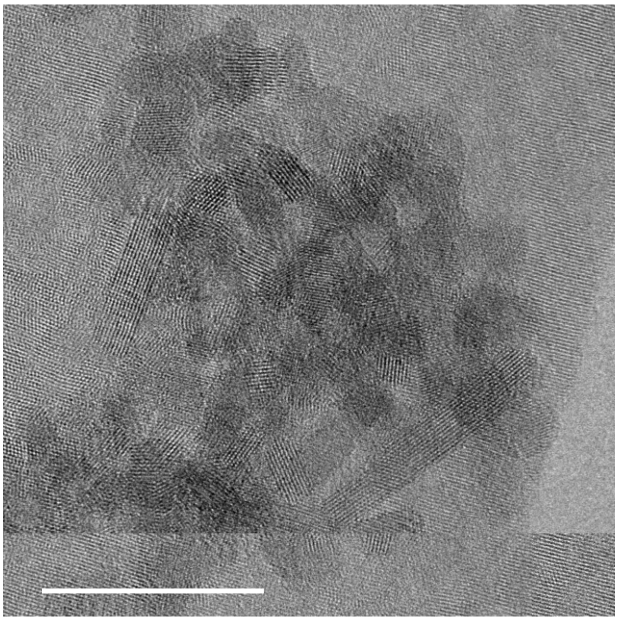

Figure S3. HRTEM image of $SnO_2/ZnSe(N_2H_4)_{0.5}$ heterojunction nanocomposites with a $R_{Sn/Zn}$ value of 0.10.

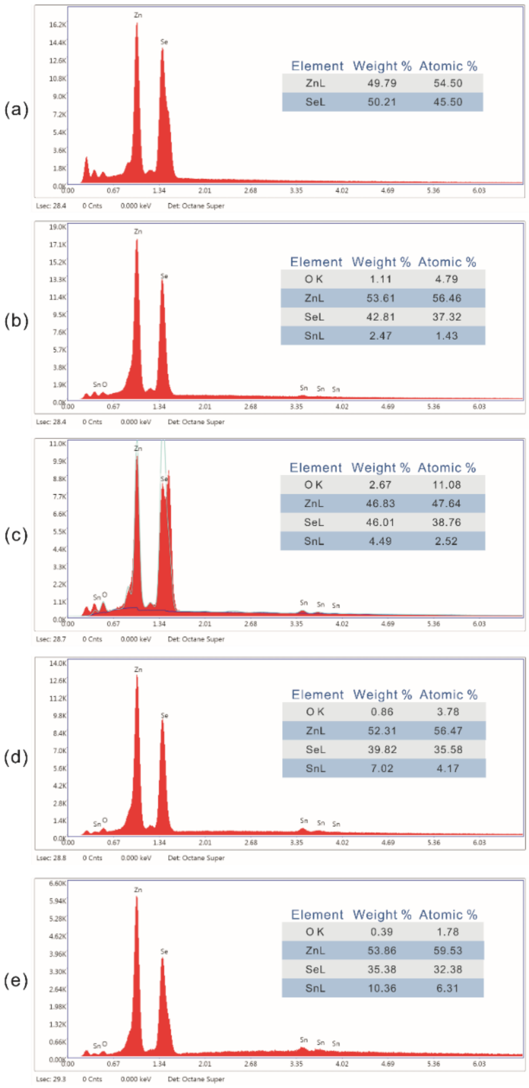

Figure S4. EDX spectra of SnO_2/ZnSe$(N_2H_4)_{0.5}$ heterojunction nanocomposites having $R_{Sn/Zn}$ = 0.00 (a), 0.03 (b), 0.05 (c), 0.08 (d), and 0.10 (e) at sample areas of 15 μm × 15 μm on copper grids. The calculated atomic ratios of [Sn]-to-[Zn] are 0.00 (a), 0.03 (b), 0.05 (c), 0.07 (d), and 0.11 (e).

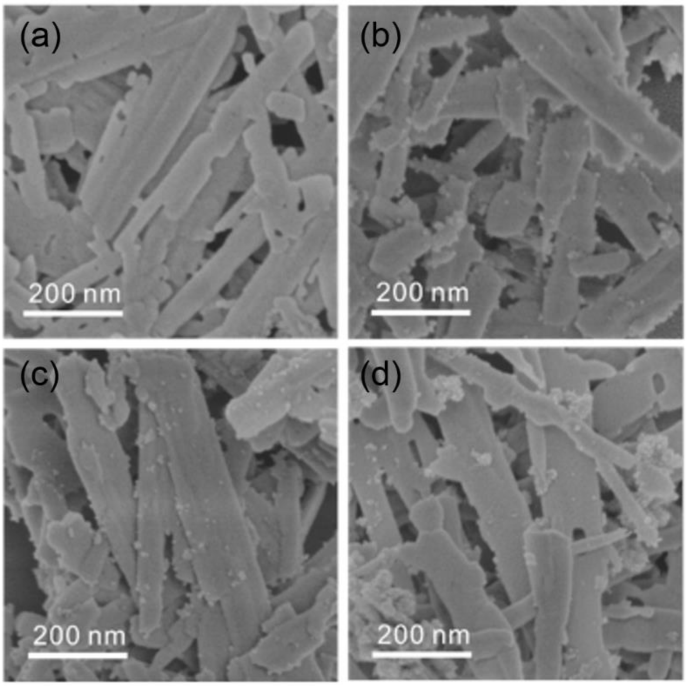

Figure S5. SEM images of SnO_2/ZnSe heterojunction nanocomposites having $R_{Sn/Zn}$ = 0.00 (a), 0.05 (b), 0.08 (c), and 0.10 (d).

Table S1. BET surface areas, average pore sizes, and catalytic degradation rate constants of RhB *via* nanocatalysts under visible-right irradiation of a 300 W Xe lamp.

Catalyst type	$R_{Sn/Zn}$	BET surface area (m^2/g)	Average pore size (nm)	Total pore volume (cm^3/g^{-1})	k (min^{-1})
P25					2.27×10^{-3}
SnO_2 nanoparticle	∞				2.83×10^{-4}
ZnSe nanoparticle	0.00				4.12×10^{-3}
$ZnSe(N_2H_4)_{0.5}$ nanobelts	0.00	24.1	26.9	0.16	1.41×10^{-2}
Nanocomposites	0.03	28.9	19.3	0.14	2.93×10^{-2}
Nanocomposites	0.05	29.6	19.4	0.14	3.82×10^{-2}
Nanocomposites	0.08	31.5	22.1	0.17	4.65×10^{-2}
Nanocomposites	0.10	31.9	16.2	0.13	3.98×10^{-2}
Nanocomposites	0.12	30.4	18.3	0.14	3.41×10^{-2}

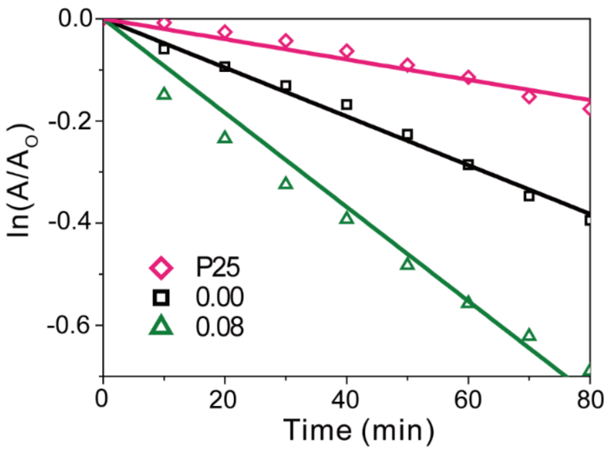

Figure S6. First-order decay profiles of RhB degradation through TiO_2 (P25 from Degussa), and $SnO_2/ZnSe(N_2H_4)_{0.5}$ heterojunction nanocomposites having $R_{Sn/Zn} = 0.00$, $R_{Sn/Zn} = 0.08$.

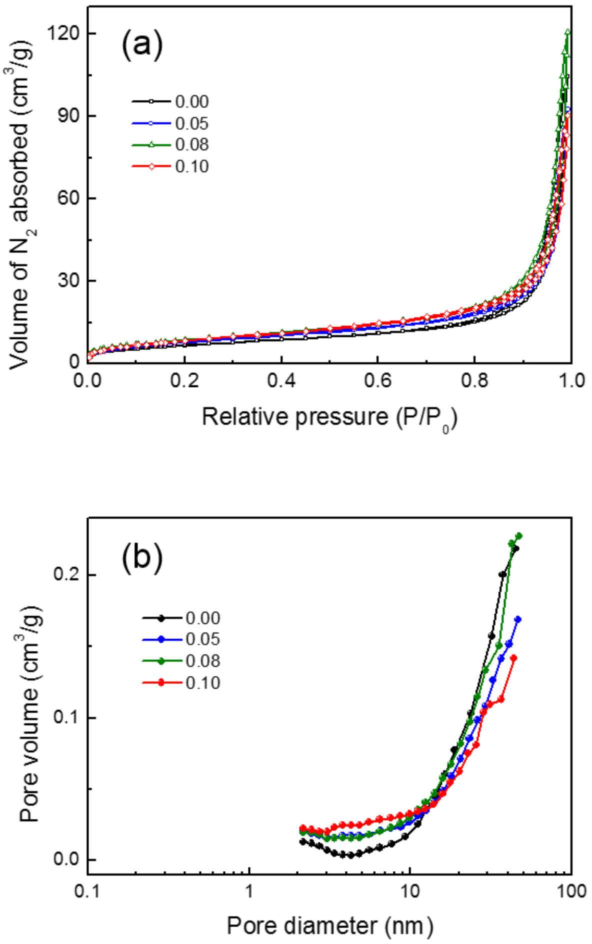

Figure S7. Nitrogen adsorption-desorption isotherms (a) and pore-size distributions (b) of $SnO_2/ZnSe(N_2H_4)_{0.5}$ heterojunction nanocomposites with indicated $R_{Sn/Zn}$ values.

Table S2. Catalytic degradation rate constants of RhB via $R_{Sn/Zn}$ = 0.08 nanocatalysts under visible-right irradiation with various hole scavengers.

Hole scavengers	$R_{Sn/Zn}$	k (min^{-1})	relative k
none	0.08	4.23×10^{-2}	1
ammonium oxalate (AO)	0.08	3.52×10^{-2}	0.832
Benzoquinone (BQ)	0.08	2.30×10^{-3}	0.054
Isopropanol (IPA)	0.08	3.39×10^{-3}	0.080

(a) $RhB + hv(\text{visible light}) \rightarrow RhB^{*}$

$RhB^{*}(e^{-}) + SnO_2/ZnSe(N_2H_4)_{0.5} \rightarrow RhB^{*+} + SnO_2/ZnSe(N_2H_4)_{0.5}(e^{-})$

(b) $SnO_2/ZnSe(N_2H_4)_{0.5}(e^{-}) + O_2 \rightarrow SnO_2/ZnSe(N_2H_4)_{0.5} + O_2^{-\bullet}$

$2\ SnO_2/ZnSe(N_2H_4)_{0.5}(e^{-}) + 2H^{+} + O_2 \rightarrow H_2O_2$

$SnO_2/ZnSe(N_2H_4)_{0.5}(e^{-}) + H_2O_2 \rightarrow OH^{-} + OH^{\bullet}$

$H_2O_2 + O_2^{-\bullet} \rightarrow OH^{-} + O_2 + OH^{\bullet}$

(c) $RhB^{*+} \rightarrow$ N-deethylation $\rightarrow$ Mineralization

$RhB + OH^{\bullet} \rightarrow$ Mineralization

Figure S8. Mechansim of photocatalytic activity in rhodamine B with $SnO_2/ZnSe(N_2H_4)_{0.5}$ system.

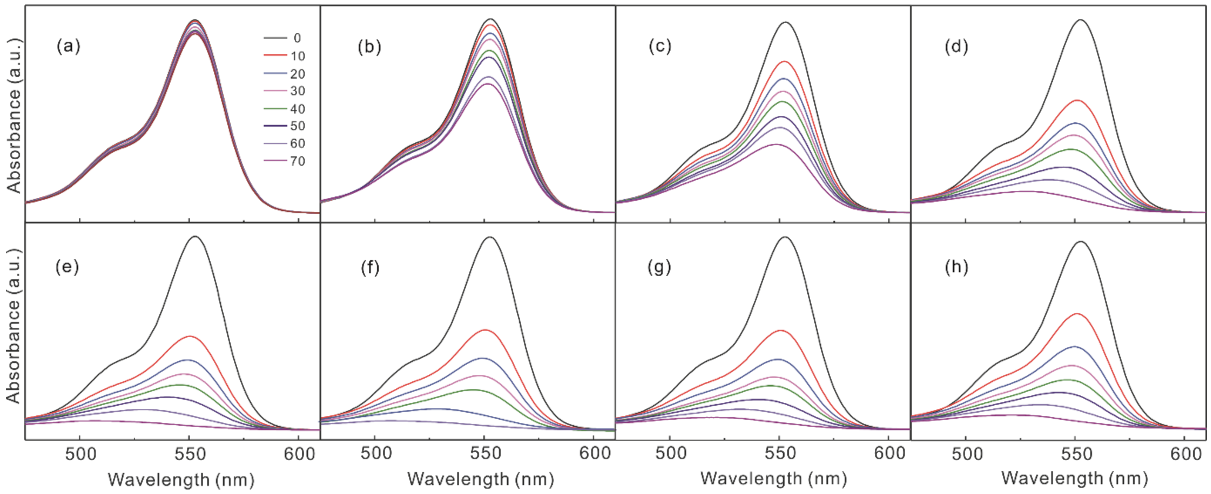

Figure S9. Absorption spectra of RhB solutions in the presence of light without catalysts (a), with commercial ZnSe (b), with $SnO_2/ZnSe(N_2H_4)_{0.5}$ heterojunction nanocomposites having $R_{Sn/Zn}$ = 0.00 (c), 0.03 (d), 0.05 (e), 0.08 (f), 0.10 (g), and 0.12 (h), measured at elapsed times indicated in the units of min.

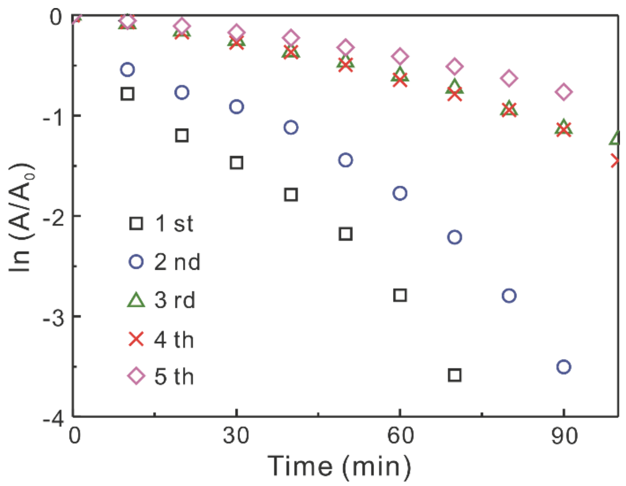

Figure S10. Reusability test of $SnO_2/ZnSe(N_2H_4)_{0.5}$ ($R_{Sn/Zn}$ = 0.08) heterojunction nanocomposites *via* repeated photocatalytic RhB-degradation experiments.

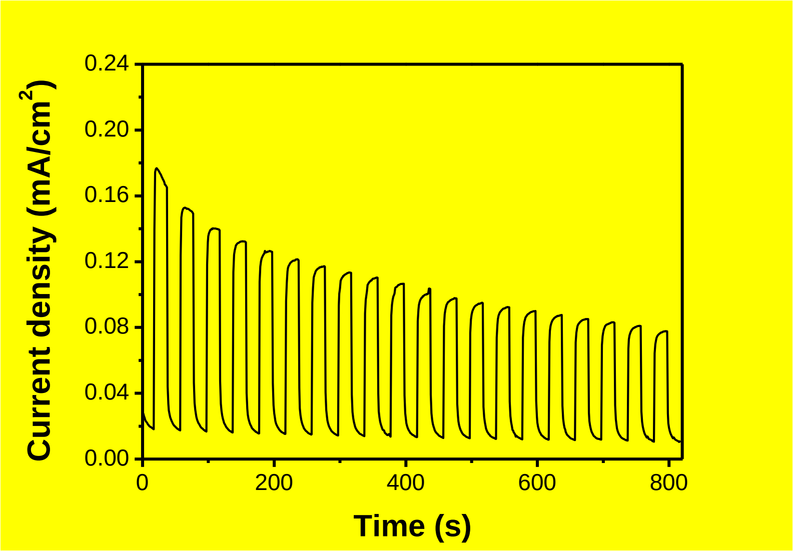

Figure S11. Transient photocurrent response curve of $SnO_2/ZnSe(N_2H_4)_{0.5}$ ($R_{Sn/Zn}$ = 0.08) heterojunction nanocomposites.

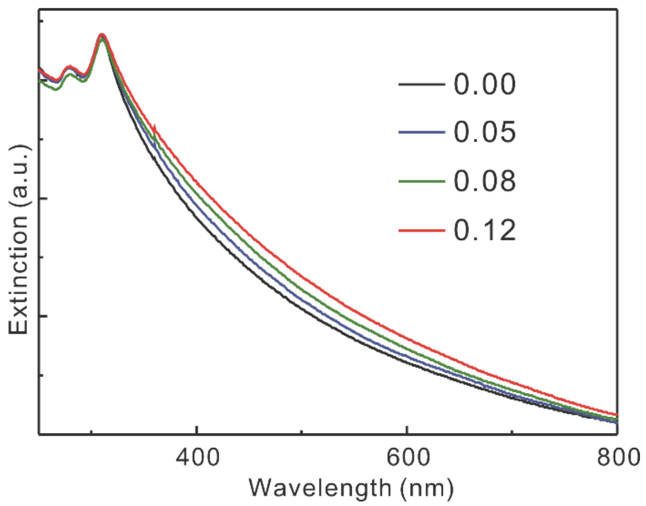

Figure S12. Extinction spectra of $SnO_2/ZnSe(N_2H_4)_{0.5}$ heterojunction nanocomposites with indicated $R_{Sn/Zn}$ values.

Table S3. Emission decay kinetic constants of $SnO_2/ZnSe(N_2H_4)_{0.5}$ heterojunction nanocomposites with indicated $R_{Sn/Zn}$ values suspended in ethanol. Note that fast components are thought as laser peaks, which correspond to instrument response function (IRF).

$R_{Sn/Zn}$	Relative PL intensity	Decay time (ps)
0.00	1.00	240 (75%) + 1600 (25%)
0.05	0.53	240 (75%) + 1440 (25%)
0.08	0.33	240 (75%) + 1220 (25%)
0.10	0.14	240 (75%) + 1000 (25%)

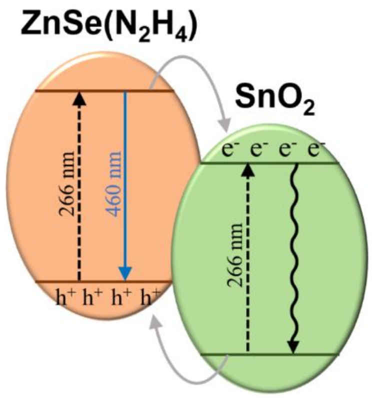

Figure S13. Schematic drawing of charge separation mechanism investigated by the spectroscopic method of the $SnO_2/ZnSe(N_2H_4)_{0.5}$ heterojunction nanocomposites.
